# Perinatal Oxidative Stress and Kidney Health: Bridging the Gap between Animal Models and Clinical Reality

**DOI:** 10.3390/antiox12010013

**Published:** 2022-12-21

**Authors:** You-Lin Tain, Chien-Ning Hsu

**Affiliations:** 1 Department of Pediatrics, Kaohsiung Chang Gung Memorial Hospital, Kaohsiung 833, Taiwan; 2 College of Medicine, Chang Gung University, Taoyuan 333, Taiwan; 3 Department of Pharmacy, Kaohsiung Chang Gung Memorial Hospital, Kaohsiung 833, Taiwan; 4 School of Pharmacy, Kaohsiung Medical University, Kaohsiung 807, Taiwan

**Keywords:** oxidative stress, antioxidant, kidney disease, nitric oxide, asymmetric dimethylarginine, reactive oxygen species, melatonin, fetal programming

## Abstract

Oxidative stress arises when the generation of reactive oxygen species or reactive nitrogen species overwhelms antioxidant systems. Developing kidneys are vulnerable to oxidative stress, resulting in adult kidney disease. Oxidative stress in fetuses and neonates can be evaluated by assessing various biomarkers. Using animal models, our knowledge of oxidative-stress-related renal programming, the molecular mechanisms underlying renal programming, and preventive interventions to avert kidney disease has grown enormously. This comprehensive review provides an overview of the impact of perinatal oxidative stress on renal programming, the implications of antioxidant strategies on the prevention of kidney disease, and the gap between animal models and clinical reality.

## 1. Introduction

Imbalances between reactive oxygen species or reactive nitrogen species (ROS and RNS, respectively) and innate antioxidant systems result in oxidative stress [1]. During pregnancy, ROS and RNS have dual roles in fetal development [2]. Normally, a moderate increase in ROS and RNS levels is essential for placental angiogenesis, cell differentiation, and fetal organogenesis. In contrast, the overproduction of ROS and RNS, as observed in compromised pregnancies, is associated with adverse pregnancy and fetal outcomes [3]. In addition, a surplus of ROS reduces nitric oxide (NO) bioavailability. NO is recognized as a key regulator of both maternal and fetal homeostasis during gestation [4]. 

After birth, newborns are highly vulnerable to ROS- and RNS-induced oxidative damage [5]. A newborn encounters the transition from a hypoxic intrauterine environment to a postnatal oxygen-rich environment with an approximately five-fold increase in oxygen exposure. Notably, preterm babies have increased susceptibility to increased oxidative stress conditions (e.g., infection and inflammation), in addition to their antioxidant defenses being impaired [6].

During development, the kidneys are vulnerable to oxidative stress and other environmental insults that impair nephrogenesis [7]. In humans, kidney development starts at week three and is completed at week 36 of pregnancy [8]. An exponential increase in nephrons occurs at 18–32 weeks of pregnancy. Nephron development is complete at the end of gestation [9]. Thus, preterm birth is associated with a reduction in nephron numbers and increased risk of kidney disease [9]. Impaired nephrogenesis results in low nephron endowment and a spectrum of defects in the kidneys and urinary tract [10].

To date, little information is available about the influence of perinatal oxidative stress on the development of kidney disease in humans. Unlike humans, nephrogenesis in rats lasts after birth and finishes at 1–2 weeks postnatally [11]. Developing kidneys are mostly vulnerable to suboptimal pre-, peri-, and early postnatal conditions, resulting in alterations in structure and function, namely renal programming [12]. A growing body of evidence from animal models has offered greater understanding of the link between oxidative stress and the development of kidney disease [7,13,14]. On the contrary, data from animal studies indicated that the perinatal use of antioxidants was able to reverse programming processes and prevent kidney diseases of developmental origins [15].

Although substantial progress has been achieved in developing various animal models to study oxidative-stress-related renal programming, the need for meaningful translation into clinical practice is still a research priority. Hence, the present review seeks to highlight the best available evidence on the interplay between perinatal oxidative stress and renal programming. We attempt to discuss the impact of oxidative stress on fetuses and neonates, its associations with common mechanisms behind renal programming, and the potential of antioxidant strategies for the prevention of kidney disease.

## 2. Oxidative Stress and Fetal Programming

### 2.1. ROS, RNS, and NO

**Figure 1:**
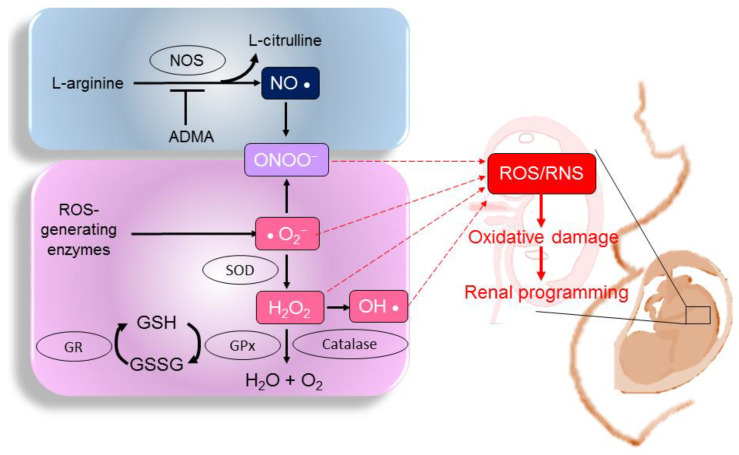
Diagram illustrating the pathways that generate reactive oxygen species (ROS) and reactive nitrogen species (RNS), the nitric oxide (NO) pathway, and antioxidant systems in a fetus. The overproduction of ROS or RNS under adverse intrauterine conditions overwhelms the antioxidant system, resulting in oxidative damage and, thereby, compromising renal development. NOS: nitric oxide synthase; ADMA: asymmetric dimethylarginine; ONOO^−^: peroxynitrite; SOD: superoxide dismutase; GPx: glutathione peroxidase; GR: glutathione reductase; GSH: reduced glutathione; GSSH: oxidized glutathione; H_2_O_2_: hydrogen peroxide; OH•: hydroxyl radical.

The disequilibrium of the pro-oxidant–antioxidant balance leads to oxidative stress. ROS and RNS can be radical or non-radical compounds. Examples of ROS include free radicals, such as superoxide anions (•O_2_^−^) and hydroxyl radicals (•OH), as well as non-radicals, such as hydrogen peroxide (H_2_O_2_) [16]. Nitrogen-containing oxidants, such as nitric oxide (•NO), nitrogen dioxide (NO_2_), and peroxynitrite (ONOO^−^), are called RNS [17]. On the other hand, the excess of ROS or RNS can be neutralized by antioxidant systems including enzymatic components (e.g., superoxide dismutase (SOD) and non-enzymatic antioxidants (e.g., glutathione) [18]. NO is generated by NO synthases (NOSs) [19]. Asymmetric and symmetric dimethylarginine (ADMA and SDMA, respectively) can uncouple NOSs to generate peroxynitrite, further reducing NO bioavailability and enhancing oxidative stress [20]. As ROS, RNS, and NO are essential for pregnancy, maintenance of their balance is crucial for the normal development of a fetus. The ROS- and RNS-generating pathways, the NO pathway, and antioxidant systems in a fetus, as well as their interconnections with renal programming, are illustrated in Figure 1.

### 2.2. Studies in Humans: Oxidative Stress in Fetuses and Neonates

A fetus obtains sufficient amounts of oxygen to meet its growth and metabolic needs. During the first trimester, the fetal oxygen requirement is low. Nevertheless, increasing oxygen levels are required for the establishment of fetal–placental circulation and the rapid fetal weight gain in the second and third trimesters [21]. Although a moderate physiological level of ROS is crucial to maintain a healthy pregnancy [2,3], prior work has indicated that increased oxidative stress exists in a variety of complications in pregnancy. These include, but are not limit to, gestational diabetes [22], preeclampsia [23], preterm birth [24], placenta dysfunction [25], maternal obesity [26], preterm premature rupture of membranes [27], and intrauterine growth retardation (IUGR) [28]. Fetuses with these complications during pregnancy have long-term consequences in their health later in life.

Preterm babies are particularly vulnerable to oxidative damage due to the immaturity of antioxidant systems, high non-protein-bound iron (NPBI) levels, and the high energy requirements for growth [5]. These instances of oxidative damage include the oxidation of biological molecules, such as lipids, proteins, and DNA. The plasma of preterm babies showed reduced antioxidant capacity characterized by low levels of SOD, CAT, GPX, copper, vitamin E, selenium, ceruloplasmin, zinc, etc. [29]. Oxidative stress is identified as one of the main causes responsible for several complications of prematurity, including necrotizing enterocolitis (NEC), bronchopulmonary dysplasia (BPD), retinopathy of prematurity (ROP), intraventricular hemorrhage (IVH), respiratory distress syndrome (RDS), kidney disease, etc. [5].

### 2.3. Biomarkers of Oxidative Stress in Clinical Practice

Oxidative stress in fetuses and neonates has been evaluated by assessing products of lipid peroxidation in the serum or amniotic fluid, such as malondialdehyde (MDA), F2-isoprostanes (F2-IsoPs), 4-hydroxy-2-nonenal (4-HNE), and thiobarbituric-acid-reactive substances (TBARSs) [30,31]. In addition, oxidative-stress-related protein damage can be measured by advanced oxidation protein products (AOPPs) in the serum or cord blood [30,32]. Regarding DNA damage, 8-hydroxy-2′-deoxyguanosine (8-OHdG) is a commonly used biomarker, as it is an oxidized nucleoside released upon the repair of damaged DNA [33]. 8-OHdG is excreted in urine without further metabolism; therefore, urinary 8-OHdG is employed as a biomarker of oxidative stress in newborn medicine [34]. The ratio of reduced to oxidized glutathione (GSH/GSSG) is another biomarker employed [35], as it represents a dynamic balance between oxidants and antioxidants. Moreover, measurements of antioxidant status that include the total antioxidant capacity (TAC) and antioxidant enzymes (e.g., SOD and catalase) can also be utilized as oxidative stress biomarkers [36].

Regarding RNS, plasma and cerebrospinal fluid levels of 3-nitrotyrosine have been applied as markers for peroxynitrite in neonates [37,38]. ADMA and the NO metabolites of nitrite and nitrate have been measured in the plasma and urine [39,40]. Preeclampsia is connected to low NO bioavailability, represented by the L-arginine-to-ADMA ratio [41]. Thus far, NO can be detected in vivo using various methods, such as chemiluminescence, fluorescence, and electron spin resonance spectroscopy. Nevertheless, NO measurements by these methods are still limited in neonatal medicine.

### 2.4. What Is Missing from Human Studies?

At full-term birth, neonates generally possess a complete endowment of nephrons. Nevertheless, nephron numbers may be reduced in infants who are born preterm due to compromised pregnancy, inadequacy of postnatal nutrition, intrauterine growth retardation (IUGR), and treatment with certain medications (e.g., gentamicin) after birth [42]. Low nephron numbers play a part in glomerular hypertension and hyperperfusion injury, consequently provoking a vicious cycle of more nephron loss later in life [43]. Importantly, low nephron endowment presumably enacts a first hit to the kidneys, which makes the remaining glomeruli more vulnerable to developing CKD when facing second-hit kidney injuries [44].

To date, nephron numbers cannot be calculated in vivo. Despite average nephron numbers reported at about 1 million in each kidney based on prior studies of kidney autopsies, human nephron numbers are highly variable (10-fold difference) [8]. In human studies, there remain unmet needs to elucidate the molecular mechanisms behind perinatal oxidative-stress-induced kidney disease and to develop interventions necessary to prove causation. Clinically, kidney biopsies are a technically difficult procedure in children, especially in neonates. It should be noted that it remains largely unknown whether there is a link between kidney pathologies and circulating oxidative stress biomarkers in fetuses and neonates. This is the reason why much of our knowledge of oxidative-stress-related renal programming, the molecular mechanisms underlying renal programming, and preventive interventions to avert kidney disease mainly originate from animal studies.

## 3. Animal Models of Oxidative-Stress-Related Renal Programming

Through the use of animal models, our understanding of the molecular mechanisms behind renal programming has grown enormously in recent years [7,12]. Core mechanisms include, but are not limited to, oxidative stress, NO deficiency, low nephron number, aberrant activation of the renin–angiotensin system, dysregulated nutrient-sensing signals, and gut microbiota dysbiosis [7,12,45,46]. The tight interconnections between oxidative stress and other common mechanisms behind renal programming mean that oxidative stress plays a prominent role.

Table 1 provides a summary of animal models of oxidative-stress-related renal programming [33,35,47,48,49,50,51,52,53,54,55,56,57,58,59,60,61,62,63,64,65,66,67,68,69,70,71,72,73,74,75,76,77]. The current review is chiefly restricted to adverse environmental cues beginning in gestation and lactation. A wide range of environmental cues can lead to oxidative-stress-related renal programming, including imbalanced maternal nutrition [47,48,49,50,51,52,53,54,55,56,57,58,59], maternal disorders [60,61,62,63,64,65,66,67,68,69], environmental chemical and toxin exposure [70,71,72,73], and medication use [74,75,76,77].

### 3.1. Maternal Insults

Table 1 illustrates that nutritional imbalance is the most common insult that induces renal programming. Types of maternal nutritional insults can be grouped into different models that aim to reduce calorie intake [47,48], reduce protein intake [49], increase fructose intake [50,51], manipulate methyl donor [52] or iron intake [53], and increase fat intake [54,55,56,57,58,59]. In addition, maternal disorders, such as NO deficiency [60,61], diabetes [62,63], preeclampsia [64], CKD [65,66], reduced uterine perfusion [67], hypertension [68], and inflammation [69], have all been reported to impair nephrogenesis, resulting in renal programming. Environmental toxins, such as bisphenol A [70], 2,3,7,8-tetrachlorodibenzo-p-dioxin (TCDD) [71], di-n-butyl phthalate [72], and smoking [73], also contribute to renal programming. Moreover, medications, such as glucocorticoid, are able to induce renal programming [74,75,76,77].

As all nutrients during pregnancy have essential roles in fetal development, the excessive or insufficient intakes of certain nutrients have been employed to establish animal model for studying renal programing. As shown in Table 1, different maternal nutritional insults could induce the same phenotype of hypertension, suggesting there might be common mechanisms involved in nutritional programming [78]. Conversely, the BP of offspring exposed to high-fat maternal diets could vary according to age, sex, fatty acid composition, and strain [79]. In addition, the data from Table 1 indicate that high-fat maternal diets could induce renal programming related to various sources and mechanisms of oxidative stress. Accordingly, a deeper understanding of oxidative-stress-induced nutritional programming may help to limit or avoid specific foods during pregnancy and develop effective nutritional interventions for clinical practice.

As shown in Table 1, rats are the preferred animals used to study renal programming, followed by mice and sheep. Unlike humans, kidney development in rats lasts until 1–2 week after birth [11]. Adverse environmental conditions, not only during gestation but also lactation, can affect kidney development, consequently leading to kidney disease later in life. As each rat month is roughly equivalent to three human years in adulthood [80], Table 1 illustrates the ages at evaluation, allowing calculations for reference to human ages.

Table 1 shows that the most common outcome of renal programming evaluation is hypertension. Although several environmental cues have been connected to low nephron endowment [81], the interconnection between low nephron number and oxidative stress has only been reported in models of streptozotocin-induced diabetes [62], caloric restriction [47], and maternal smoking exposure [73]. In addition to reduced nephron number, renal hypertrophy [47,48,58], glomerulosclerosis [59], tubulointerstitial injury [47,48,62,68,72,74], renal dysfunction [57,74], and albuminuria [58,59,73] are major adverse renal outcomes associated with renal programming (Table 1).

### 3.2. Oxidative-Stress-Mediated Mechanisms

As a fetus has low antioxidant capacity, a surplus of ROS or RNS under adverse intrauterine conditions can overwhelm antioxidants, resulting in oxidative damage and, thereby, compromising fetal development [2,3]. Cumulative evidence supports the key role of oxidative stress implicated in fetal programming. ROS can mediate several key epigenetic processes, such as DNA methylation, histone modifications, and micro-RNAs (mRNAs) [82]. It is noteworthy that these epigenetic modifications of genes are considered crucial mechanisms for fetal programming [83].

NO is also involved in epigenetic regulation and fetal programming [84,85]. ADMA can reduce NO production and increase ROS [20]. In our prior work, ADMA-treated embryonic kidneys exhibited reductions in nephron numbers in a dose-dependent manner [86]. We also evaluated a transcriptome analysis of developing kidneys in response to ADMA. Embryonic kidneys grown in 10 µM ADMA were isolated for a next-generation RNA sequencing (NGS) analysis, and 1221 differentially expressed genes (DEGs; 735 up- and 486 down-regulated) were identified [86]. In a model of maternal NO inhibition by N^G^-nitro-L-arginine-methyl ester (L-NAME), a total of 2289 DEGs (1259 up- and 1030 down-regulated) were identified in neonatal kidneys [60]. Among these DEGs, several genes were related to kidney development and epigenetic regulation. These observations suggest that a link between oxidative stress and epigenetic gene regulation during pregnancy could represent a strong contribution to renal programming and kidney disease risk in offspring later in life.

Renal programming can be attributed to several oxidative-stress-mediated mechanisms, including increased ROS-producing enzyme expression [59], increased ROS [68,72,73,77], increased peroxynitrite [59,63], decreased antioxidant capabilities [54,57,74], increased ADMA [47,48,62,64,65,66,70,71], reduced NO bioavailability [47,48,50,61,62,64,65,66,70,75,76,77], and increased oxidative damage [47,48,49,50,51,52,53,54,55,56,57,58,60,63,69,70,71,74,76].

**Figure 2:**
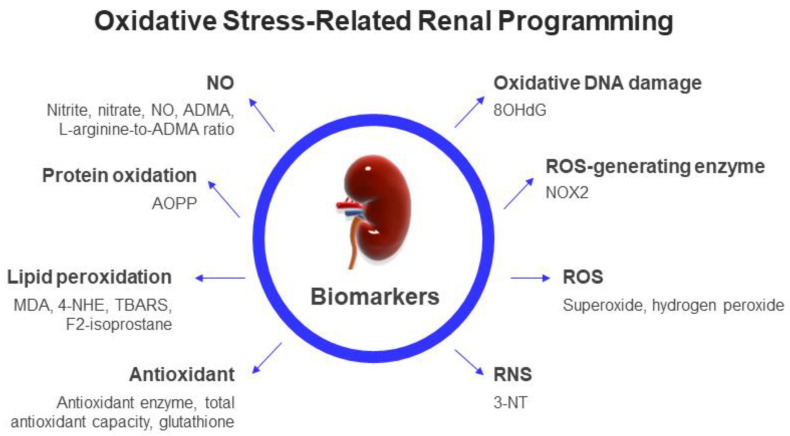
Schema summarizing the potential biomarkers regarding oxidative-stress-related renal programming in clinical and experimental studies. NO: nitric oxide; ADMA: asymmetric dimethylarginine; AOPPs: advanced oxidation protein products; 4-HNE: 4-hydroxy-2-nonenal; MDA: malondialdehyde; TBARS: thiobarbituric acid; 8-OHdG: 8-hydroxy-2′-deoxyguanosine; 3-NT: 3-nitrotyrosine.

As delineated earlier, several biomarkers of lipid peroxidation have been demonstrated in neonates, such as MDA, F2-IsoPs, and TBARS. Table 1 reveals that these biomarkers of lipid peroxidation are elevated in offspring kidneys in different rodent models of renal programming [49,54,57,60,63,66,69,74]. In addition, 8-OHdG, a frequently studied oxidative DNA damage marker, is highly expressed in rat offspring kidneys and is correlated to adverse renal outcomes [47,48,50,51,52,53,55,56,58,65,66,70,71,76]. As ROS is difficult to determine in human kidneys, animal studies have provided evidence that increased renal ROS is associated with adverse renal outcomes in models of reduced uterine perfusion [73], maternal angiotensin II administration [68], maternal DEHP exposure [72], and maternal smoking exposure [73]. Renal 3-NT level can also be used to detect peroxynitrite in rat models of renal programming [59,63]. Moreover, numerous studies in Table 1 indicate that an impaired ADMA/NO pathway contributes to oxidative-stress-induced renal programming [47,48,62,64,65,66,70,71,75,76,77]. In summary, these observations support the idea that oxidative-stress-induced renal programming contributes to adverse renal outcomes later in life. The impact of oxidative stress on renal programming can be evaluated by biomarkers that quantify the levels of ROS, RNS, NO, antioxidants, and oxidation by-products from DNA, protein, and lipid damage, as illustrated in Figure 2.

## 4. Antioxidant Strategies for Kidney Health

As mentioned above, perinatal oxidative stress plays a pivotal role in renal programming, resulting in adult-onset kidney diseases. It is reasonably assumed that a surplus of ROS or RNS may be amenable to antioxidant therapies, which, if administered in early life, may avert the development of kidney diseases. Even though the role of oxidative stress in the pathogenesis of renal programming is undoubted, the positive effects of antioxidant therapies on kidney diseases remain inconclusive clinically [87,88], as well as in fetuses and neonates [89,90,91]. While the majority of human trials have not confirmed any evidence of kidney benefits from antioxidant supplementation, we recognize the potential benefit of antioxidant therapies through current evidence in preclinical animal models and limited human studies.

Antioxidants can be grouped as enzymatic or non-enzymatic and natural or synthetic. They are categorized by mechanism of action as either targeting ROS or NO. As reviewed elsewhere [7], data from animal studies indicate that the uses of several natural antioxidants, including vitamins, amino acids, melatonin, and polyphenol, during pregnancy and lactation have shown to benefits to kidney health and prevent renal programming. Sources of natural antioxidants are mainly plants, i.e., vegetables, fruits, seeds, and nuts, which are rich in vitamins, polyphenol, carotenoids, and glutathione. Along with natural antioxidants, some synthetic antioxidants have also been implemented in animal models of renal programming.

**Figure 3:**
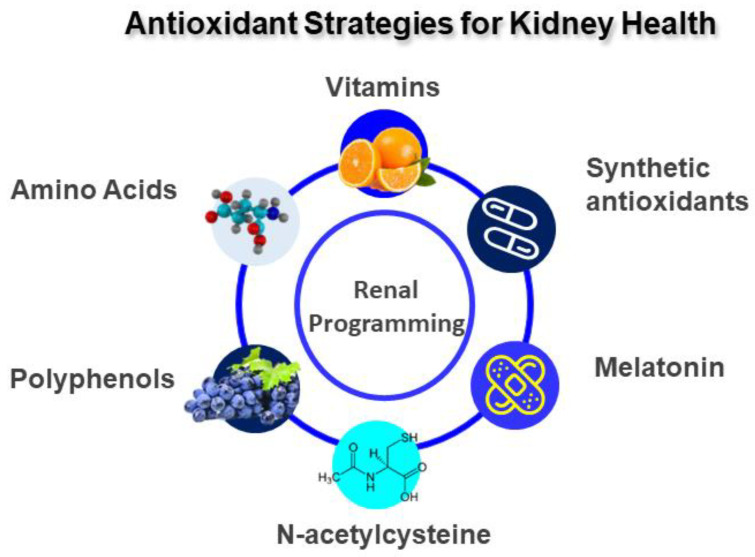
Diagram outlining a potential antioxidant strategy to prevent adult-onset kidney diseases.

As mentioned earlier, nutritional programming is emerging as a critical mechanism contributing to oxidative-stress-related renal programming. It is noteworthy that nutritional programming can also be advantageous [92]. Several nutritional interventions with antioxidant or anti-inflammatory diets have been proved effective in preventing the development of adult-onset kidney diseases with the use of animal models [7] (Figure 3). These are discussed below.

### 4.1. Vitamins

Vitamins A, C, and E, as well as selenium, folic acid, etc., exhibit advantageous effects on kidney health [93]. The most frequently used antioxidant supplements are vitamins C and E. Vitamin C, a water-soluble antioxidant, is a scavenger of free radicals and a reducing agent [94]. Vitamin E, a lipid-soluble antioxidant, inhibits several oxidative enzymes to reduce ROS production [95].

Vitamin C or E supplementation alone during pregnancy protects maternal lipopolysaccharide (LPS)-exposure-primed offspring hypertension, a major phenotype of renal programming [96,97]. Additionally, the combined supplementation of vitamins C and E with selenium and folic acid averted offspring hypertension in a rat model of maternal caloric restriction [98].

Though several vitamins exhibit beneficial effects on oxidative-stress-related kidney diseases [93], little attention has been paid to determining their protective actions starting at the fetus and neonate stages. As mentioned earlier, the disturbance of epigenetic regulation can lead to oxidative stress, linking to renal programming. Although vitamins B6, vitamin B12, and folate contribute to DNA methylation and have recognized roles as methyl donors [99], it would be interesting to know whether perinatal use of these vitamins can prevent renal programming via the regulation of epigenetics.

On the other hand, a meta-analysis recruiting 56 clinical trials concluded that high doses of vitamin A, β-carotene, and vitamin E appeared to increase mortality [100]. It is noteworthy that excessive dietary vitamin A intake was linked to human birth defects [101]. Perinatal vitamin supplements should only be administered in cases of deficiency, not as a usual intake. The contamination of vitamin supplements is another concerning problem being discussed. With the particular vulnerability of a developing fetus, attention to the detrimental effects of heavy metals and toxic elements contaminating vitamins consumed in pregnancy is imperative [102].

### 4.2. Amino Acids

The moderation of dietary amino acid consumption has therapeutic and protective effects on kidney diseases [103]. Several amino acids are known to possess antioxidant properties [104].

l-arginine is a substrate for the NOS production of NO, and l-citrulline is a precursor of l-arginine [105,106]. Considering that NO deficiency is a major pathogenetic mechanism behind renal programming, perinatal use of these two amino acids has been assessed to protect offspring against adult kidney diseases [105,106].

Human kidneys can covert l-citrulline to l-arginine [106]. Oral l-citrulline supplementation enables bypassing hepatic metabolism to enhance l-arginine production and raise NO levels [106]. Currently, maternal l-citrulline supplementation has been reported to enhance NO bioavailability and protect adult rat offspring against renal programming in oxidative-stress-related models of streptozotocin-induced diabetes [62], maternal caloric restriction [47], and prenatal dexamethasone exposure [75].

Additionally, L-tryptophan and L-cysteine have also been assessed as reprogramming interventions to target oxidative stress in maternal CKD-primed renal programming models [65,107]. Despite other amino acids, such as L-taurine and branched-chain amino acids, showing beneficial potential for kidney diseases [108], whether their protective effects are attributed to the reduction of oxidative stress awaits further clarification.

### 4.3. Melatonin

Melatonin is an endogenous tryptophan-derived indolamine with multiple biofunctions [109]. Melatonin plays an essential role in pregnancy and fetal development [110]. Melatonin and its metabolites are able to scavenge ROS and RNS, upregulate antioxidant enzymes, and increase NO bioavailability [111,112]. Hence, it has been clinically applied as an antioxidant therapy in pregnant women and neonates [113,114].

Several human studies reported that melatonin treatment ameliorated oxidative stress in newborns with asphyxia, sepsis, or other conditions with overproduction of ROS [114]. Moreover, the urinary excretion of melatonin’s metabolite could be used as a biomarker for babies with IUGR, suggesting the impact of the melatonin pathway in fetal programming [115].

Data from animal studies indicated that perinatal melatonin treatment could serve as a preventive intervention for many adult-onset diseases, including kidney diseases [116]. Maternal melatonin treatment has shown kidney benefits in several models of oxidative stress programming, such as caloric restriction [48], methyl donor diet [52], maternal l-NAME exposure [60], and high fructose intake [117]. When targeting oxidative stress, the protective effects of melatonin include reduced lipid peroxidation [60], ADMA [48], and 8-OHdG expression [52], as well as enhanced NO [117].

Although melatonin has a favorable safety profile in the pediatric population [113,114,117], the use of melatonin in pregnant women is not yet recommended [118]. As such, perinatal use of melatonin as a preventive strategy for kidney health, especially in fetuses and neonates, still awaits further clinical translation.

### 4.4. Polyphenols

Polyphenols are well-known phytochemical antioxidants [119]. Resveratrol exerts antioxidant properties by acting as a metal chelator, a free-radical scavenger, an NOS activator, and a stimulator of antioxidant enzymes [119]. Accordingly, polyphenols have been utilized to improve kidney health [120,121]. Although polyphenols have been reported as a prophylactic therapy for neonatal hypoxia–ischemia [122], there is a relative scarcity of human studies to support its benefits on fetal and neonatal kidney health.

Polyphenols can be grouped as flavonoids and nonflavonoids [119]. As a flavonoid antioxidant, the use of quercetin in gestation was noted to protect adult rat progeny against high-fat maternal-diet-induced renal programming and hypertension [123]. Another example is epigallocatechin gallate. Its use in gestation and lactation moderated prenatal dexamethasone-exposure-primed hypertension in a rat model [124].

Resveratrol is a naturally occurring nonflavonoid polyphenol [125]. Its antioxidant effects include scavenging ROS and RNS, enhancing antioxidant enzymes, increasing glutathione levels, upregulating NOS expression, etc. [126]. Several rat models of renal programming, such as high-fructose diet [51], maternal ADMA administration [61], adenine-induced CKD [66], bisphenol A exposure [70], and TCDD exposure [71], have shown beneficial effects of resveratrol on renal outcomes in adult progeny. For example, perinatal resveratrol therapy could protect offspring against renal programming, accompanied by reducing renal 8-OHdG expression and increasing NO [66].

One major limitation of the clinical utility of polyphenols is low bioavailability [127]. Taking into account the interindividual variability and complexity of polyphenol pharmacokinetics, future investigations are essential to better clarify the impacts of various polyphenols on kidney health, especially in fetal and neonatal medicine.

### 4.5. N-Acetylcysteine

N-acetylcysteine (NAC) is a well-known plant antioxidant [128]. In addition, NAC is a precursor to glutathione and an L-cysteine analogue that can be used for hydrogen sulfide (H_2_S) synthesis [129]. The therapeutic role of NAC in neonatal kidney disease has been shown in a rat sepsis model [130] and a porcine neonatal asphyxia model [131], despite limited human studies in this regard.

A prior study showed that perinatal NAC therapy protected rat offspring against maternal L-NAME-administration-induced renal programming, coinciding with the enhancement of renal H_2_S-generating enzyme expression and activity [60]. In another prenatal dexamethasone and postnatal high-fat diet model [76], the protective effect of NAC against oxidative stress was associated with increased plasma glutathione level and the upregulation of H_2_S-producing enzymes. Moreover, perinatal NAC therapy could avert maternal suramin-administration-induced hypertension and oxidative stress in adult rat progeny, which was involving increases of glutathione production, restoration of NO, and augmentation of the H_2_S pathway [64].

### 4.6. Synthetic Antioxidants

Along with natural antioxidants, a few synthetic antioxidants have been utilized in kidney diseases [87,88]. MitoQ, a coenzyme Q10 analogue, could reduce oxidative stress by the suppression of superoxide production and lipid peroxidation [132]. A prior study demonstrated that perinatal MitoQ treatment averted mouse adult offspring from hypertension and reduced nephron numbers and kidney injuries in a maternal smoking model [80]. Another example is dimethyl fumarate (DMF), a classical activator of nuclear factor (erythroid-derived 2)-like 2 (Nrf2) [133]. In an antenatal dexamethasone exposure and postnatal high-fat diet model, the protective actions of DMF therapy were relevant for the reduction of oxidative stress, which was represented by reductions in ADMA and 8-OHdG, as well as increasing NO [134].

Some synthetic antioxidants classified as SOD mimetics show therapeutic potential for many disorders related to oxidative stress [135]. While the gestational use of the SOD mimetic tempol was noted to reduce proteinuria and BP in adult spontaneously hypertensive rat offspring [136], none of these synthetic antioxidants have been introduced into clinical practice in neonatal medicine.

## 5. The Gap between Animal Models and Clinical Reality

In patients with CKD, oxidative stress is present in the early stages of CKD and is more exacerbated in the end stages of kidney disease. Accordingly, the exogenous intake of antioxidants has repeatedly been shown to suppress oxidative stress in CKD patients [137,138]. However, none of these antioxidants are recommended in therapeutic guidelines for CKD. While preclinical studies using animals have highlighted antioxidant strategies as an attractive approach to kidney health, their efficacy still awaits validation in clinical reality. It is, however, important to know the correct antioxidant and the correct therapeutic dose to obtain direct benefits for the human body and to not only show beneficial effects in animal studies. Further studies in large cohorts of pregnant women are required to establish causality between perinatal antioxidant supplementation and clinical hard endpoints of renal outcomes in their children.

In view of the difficulties of recruiting pregnant women and neonates for human research, the use of breastmilk as an antioxidant strategy might be a good start. It is well known that breastmilk has a powerful antioxidant composition [139]. Given that the World Health Organization recommends exclusive breastfeeding for the first 6 months [140], the antioxidant protection provided by breastfeeding against renal programming is a significant issue that warrants further study.

Another concern is the safety of antioxidant supplements. Several antioxidants might provoke oxidative stress due to their pro-oxidative properties [141]. For example, vitamin E is known not only as a potent antioxidant, but also as a harmful pro-oxidant. If there is not enough vitamin C for its regeneration, vitamin E becomes a radical when reacting with ROS [142]. Additionally, controversy around antioxidants is due to their capacity to act as pro-oxidants depending on concentration. Therefore, there is only scientific evidence that antioxidants should be supplemented solely in cases where oxidative stress is identified.

Oxidative damage in kidneys can be determined in animal models, while human studies are limited in this regard, especially in fetuses and neonates. Accordingly, antioxidant therapies may cause unexpected damage to health, as they might reach healthy tissues that have not experienced oxidative stress damage, as well as the targeted organs of the kidneys. The balance between antioxidants and ROS or RNS should be optimal, as antioxidant extremes, namely antioxidative stress, are all damaging [143].

Regardless of recent advances in developing biomarkers of oxidative stress, most of these have not yet been assessed in the context of the early prediction of adult-onset kidney diseases. Currently, an ideal oxidative stress biomarker for kidney disease does not exist, and overlaps between biomarkers are a reality [144]. A panel of biomarkers that covers the pathogenic process of kidney disease identified in animal studies might optimize the specific value of each biomarker [145]. Therefore, the introduction of a panel of oxidative stress biomarkers correlating with the extent of kidney damage for the early identification of at-risk fetuses and neonates is a practical way to bridge the gap between animal models and clinical practice. Considering the rapid development of liquid biopsy technology with respect to kidney diseases [146], the application of liquid biopsies in the rapid diagnosis of oxidative-stress-related kidney disease should become more prominent.

## 6. Conclusions and Future Perspectives

Kidney health can be improved via oxidative-stress-targeting strategies, from pregnancy to the infantile stage [45]. First, promoting an optimal prenatal environment to minimize early-life risk factors may not only promote optimal fetal development, but may even avert oxidative-stress-mediated damage to developing kidneys. Second, several antioxidant strategies have revealed promising data in animal models, and their efficacy needs future translation into human investigations. More importantly, additional studies are required to determine the correct antioxidant with the correct dosage to avert oxidative-stress-induced renal programming. Lastly, since oxidative stress is the major pathogenic mechanism behind renal programming, the development and validation of reliable oxidative stress biomarkers correlating with kidney damage and the early identification of at-risk fetuses and neonates is urgently required in pediatric care.

## Figures and Tables

**Table 1:** Animal models of oxidative-stress-related renal programming.

Animal Models	Species/ Gender	Age at Evaluation	Mechanisms of Oxidative Stress	Renal Outcomes	Ref.
	Maternal nutritional insults				
Maternal caloric restriction diet	SD rat/M	12 weeks	↑ ADMA, ↓ NO, ↑ renal 8-OHdG expression	↓nephron number, glomerular hypertrophy, ↑ tubulointerstitial injury, hypertension	[47,48]
Maternal protein restriction diet	Wistar rat/M	12 weeks	↑ F_2_-isoprostane, ↓ glutathione	↑BP	[49]
Maternal high-fructose diet	SD rat/M	12 weeks	↓ NO, ↑renal 8-OHdG expression	↑BP	[50]
Maternal plus post-weaning high-fructose diet	SD rat/M	12 weeks	↑ renal 8-OHdG expression	↑BP	[51]
Maternal methyl-deficient diet	SD rat/M	12 weeks	↑ renal 8-OHdG expression	↑BP	[52]
Maternal high-methyl-donor diet	SD rat/M	12 weeks	↑ renal 8-OHdG expression	↑BP	[52]
Maternal iron deficiency	SD rat/M	16 weeks	↑ renal 8-OHdG expression	↑BP	[53]
Maternal high-fat and high-cholesterol diet	SD rat/M and F	90 days	↓ SOD activity in M, ↑ renal MDA level in F	↑BP	[54]
Maternal plus post-weaning high-fat diet	SD rat/M and F	16 weeks	↓ NO, ↑renal 8-OHdG expression	↑BP, ↑kidney injury in M	[55,56]
Maternal high-fat and high-cholesterol diet	SD rat/M	18 weeks	↑renal MDA, ↓antioxidant enzymatic activity	hypertension, impaired renal function	[57]
Maternal high-fat diet	C57BL/6 mice/M	9 weeks	↑renal 8-OHdG expression	↑renal hypertrophy, ↑albuminuria	[58]
Maternal high-fat diet	C57BL/6 mice/M	32 weeks	↑ renal 3-NT, ↑ renal NOX2 expression	↑renal global DNA methylation, ↑albuminuria, ↑glomerulosclerosis	[59]
	Maternal disorders				
Maternal L-NAME administration	SD rat/M	12 weeks	↑ renal F_2_-isoprostane	↑BP	[60]
Maternal ADMA administration	SD rat/M	12 weeks	↓ NO	↑BP	[61]
Streptozotocin-induced diabetes	SD rat/M	12 weeks	↓ NO, ↑ ADMA	↓nephron number,↑ tuburointerstitial injury	[62]
Streptozotocin-induced diabetes	SD rat/M	12 weeks	↑ renal TBARS, ↑3-NT	↑BP, discurbed acute renal hemodynamics	[63]
Maternal suramin administration	SD rat/M	12 weeks	↓ NO, ↑ ADMA	↑BP	[64]
Maternal adenine-induced CKD	SD rat/M	12 weeks	↓ NO, ↑ ADMA, ↑ renal 8-OHdG expression,	↑BP, ↑renal hypertrophy	[65,66]
Reduced uterine perfusion	SD rat/M	16 weeks	↑ urinary F_2_-isoprostane level and renal NADPH-oxidase-dependent superoxide	↑BP	[67]
Maternal angiotensin II administration	Wistar rat/M	18 week	↑ renal ROS	↑BP, ↑tuburointerstitial injury	[68]
Prenatal LPS Exposure	Wistar rat/M	28 weeks	↑ renal MDA	↑BP	[69]
	Toxins				
Prenatal bisphenol A exposure plus high-fat diet	SD rat/M	16 weeks	↑ ADMA, ↓ NO, ↑renal 8-OHdG expression	↑BP	[70]
Prenatal dexamethasone plus TCDD exposure	SD rat/M	16 weeks	↑ renal 8-OHdG expression, ↑ ADMA	↑BP	[71]
Maternal di-n-butyl phthalate exposure	SD rat/M and F	18 months	↑ renal ROS	Renal dysplasia,↑ tuburointerstitial injury	[72]
Matenal smoking exposure	Balb/c mice/M	13 weeks	↑ renal ROS	↓nephron number,↑albuminuria	[73]
	Medication and Drugs				
Dexamethasone administration during lactation	Wistar rat/M and F	12 weeks	↑renal MDA level, ↓SOD and catalase activity	↑Tubular necrosis, renal dysfunction	[74]
Prenatal dexamethasone exposure	SD rat/M	16 weeks	↓ renal NO	↑BP	[75]
Prenatal dexamethasone exposure plus postnatal high-fat intake	SD rat/M	16 weeks	↑ renal 8-OHdG expression, ↓ NO	↑BP	[76]
Prenatal betamethasone exposure	Sheep/M and F	18 months	↓ NO,↑ ROS	↑BP	[77]

ADMA: asymmetric dimethylarginine; MDA: malondialdehyde; 8-OHdG: 8-hydroxy-2’–deoxyguanosine; 3-NT: 3-nitrotyrosine; 4-NHE: 4-hydroxynonenal; TBARS: thiobarbituric acid; NO: nitric oxide; ROS: reactive oxygen species; CKD: chronic kidney disease; LPS: lipopolysaccharide; SD: Sprague–Dawley; TCDD: 2,3,7,8-tetrachlorodibenzo-p-dioxin; L-NAME: L-N^G^-nitro arginine methyl ester; BP: blood pressure; M: male; F: female; ↑: increase; ↓: decrease.

## Data Availability

Data are contained within the article.
